# Understanding Mutations in Human SARS-CoV-2 Spike Glycoprotein: A Systematic Review & Meta-Analysis

**DOI:** 10.3390/v15040856

**Published:** 2023-03-27

**Authors:** Reetesh Kumar, Yogesh Srivastava, Pandiyan Muthuramalingam, Sunil Kumar Singh, Geetika Verma, Savitri Tiwari, Nikunj Tandel, Samir Kumar Beura, Abhishek Ramachandra Panigrahi, Somnath Maji, Prakriti Sharma, Pankaj Kumar Rai, Dinesh Kumar Prajapati, Hyunsuk Shin, Rajeev K. Tyagi

**Affiliations:** 1 Faculty of Agricultural Sciences, Institute of Applied Sciences & Humanities, GLA University, Mathura 281406, India; 2 Department of Biotherapeutics, CSIR-Institute of Microbial Technology (IMTECH), Chandigarh 160036, India; 3 Department of Genetics, The University of Texas MD Anderson Cancer Center, Houston, TX 77030, USA; 4 Division of Horticultural Science, Gyeongsang National University, Jinju 52725, Republic of Korea; 5 Department of Zoology, School of Biological Sciences, Central University of Punjab, Ghudda, Bathinda 151401, India; 6 Division of Life Sciences, Department of Biosciences, School of Basic and Applied Sciences, Galgotias University, Gautam Buddha Nagar, Greater Noida 201310, India; 7 Institute of Science, Nirma University, SG Highway, Gujarat 382481, India; 8 Department of Radiology, University of Michigan, Ann Arbor, MI 48109, USA; 9 Biomedical Parasitology and Translational-Immunology Lab, CSIR-Institute of Microbial Technology (IMTECH), Chandigarh 160036, India; 10 Department of Biotechnology, IIET, Invertis University, Bareilly 243001, India

**Keywords:** SARS-CoV-2, spike protein, COVID-19, mutations, VoC, evolution

## Abstract

Genetic variant(s) of concern (VoC) of SARS-CoV-2 have been emerging worldwide due to mutations in the gene encoding spike glycoprotein. We performed comprehensive analyses of spike protein mutations in the significant variant clade of SARS-CoV-2, using the data available on the Nextstrain server. We selected various mutations, namely, A222V, N439K, N501Y, L452R, Y453F, E484K, K417N, T478K, L981F, L212I, N856K, T547K, G496S, and Y369C for this study. These mutations were chosen based on their global entropic score, emergence, spread, transmission, and their location in the spike receptor binding domain (RBD). The relative abundance of these mutations was mapped with global mutation D614G as a reference. Our analyses suggest the rapid emergence of newer global mutations alongside D614G, as reported during the recent waves of COVID-19 in various parts of the world. These mutations could be instrumentally imperative for the transmission, infectivity, virulence, and host immune system’s evasion of SARS-CoV-2. The probable impact of these mutations on vaccine effectiveness, antigenic diversity, antibody interactions, protein stability, RBD flexibility, and accessibility to human cell receptor ACE2 was studied in silico. Overall, the present study can help researchers to design the next generation of vaccines and biotherapeutics to combat COVID-19 infection.

## 1. Introduction

**Figure 1:**
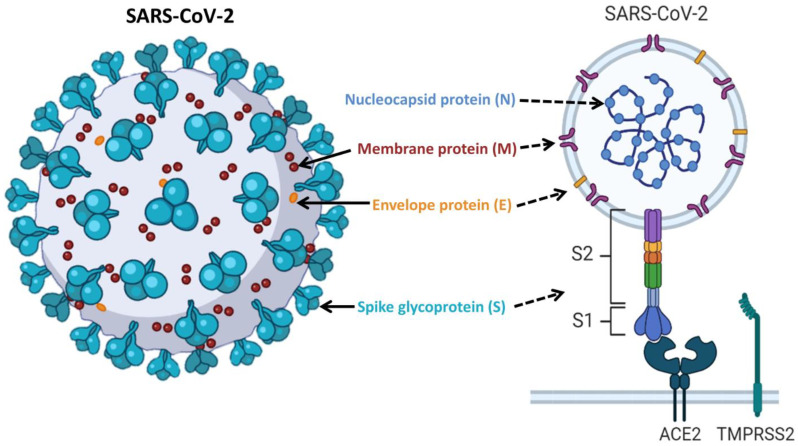
Schematic presentation illustrating the different components of SARS-CoV-2. The three-dimensional SARS-CoV-2 structure (**left panel**) shows the viral surface proteins (spikes, envelopes, and membranes) embedded in a lipid bilayer envelope. The internal structure (**right panel**), shows the corresponding protein in addition to the interior nucleocapsid protein which is associated with the single-stranded positive sense viral RNA. The interaction between the S-protein trimer of SARS-CoV-2 and the ACE2 receptor is shown in lower right. Upon binding to the receptor binding domain (RBD) of the S1 subunits, the S protein is primed with TMPRSS2 facilitating the release of the viral genome through S2-assisted fusion.

The severe acute respiratory syndrome coronavirus 2 (SARS-CoV-2) outbreak was first reported from Wuhan, China in late 2019. The disease caused by this virus is termed COVID-19, and rapidly proliferated across the world transmitted by human-to-human contact [1]. Unprecedented global transmission, morbidity, and mortality forced world health organization (WHO) to declare it a pandemic in March 2020 [2]. SARS-CoV-2 primarily infects the lower respiratory tract in humans and gives rise to the severe acute respiratory syndrome [3]. SARS-CoV-2 comprises a positive strand RNA genome that translates into four structural proteins: spike glycoprotein (S), membrane (M), envelope (E), and nucleocapsid (N) [3,4] (Figure 1). Spike protein is a fusion protein composed of two subunits, S1 and S2, and is considered the major virulence factor and antigenic determinant of SARS-CoV-2. The S1 subunit of the spike protein is responsible for receptor (human angiotensin converting enzyme 2, hACE2) binding, and S2 facilitates SARS-CoV-2 viral membrane fusion with the host cell [4,5]. The surface of the host cell also contains TMPRSS2, a serine protease that enacts S-protein priming to facilitate the entry of SARS-CoV-2 to the host [5,6]. The majority of the licensed or clinical phase vaccines and monoclonal antibodies (mAbs) against COVID-19 are based on targeting the spike protein of the virus, reflecting its relevance in viral pathogenesis [7,8,9,10,11].

SARS-CoV-2 harbors a single-stranded positive sense RNA genome of about 30 kilobase pairs [3,12]. Most RNA viruses lack 3′ exonuclease proofreading activity and possess a relatively more error-prone genome than DNA viruses. This allows the viral serodiversity to provide selective fitness for RNA viruses to cope with diverse environmental conditions and gain adaptive evolution and virulence [13]. Similar patterns of mutations have been reported in many RNA viruses: for example, A226V in E1 protein of Chikungunya virus [14]; A82V in GP protein of Ebola virus [15]; A143V and R148K in Asian avian influenza (H7N9) virus. Moreover, SARS-CoV-2 is a RNA virus, yet has an unusual proofreading activity because of its nsp14 exonuclease, enabling it to proliferate easily and disseminate globally [16].

The present study was designed to perform comprehensive in silico analyses of significant mutations in spike protein across different geographic regions over time, using the Nextstrain web server (https://nextstrain.org/ncov; accessed on 1 March 2023) and reported literature. We selected fifteen different mutations, namely A222V, N439K, N501Y, L452R, Y453F, E484K, K417N, T478K, L981F, L212I, N856K, T547K, G496S, and Y369C, based on their emergence, transmission, spread, global entropic score, and their location on the RBD domain of the spike protein. The probable consequences of these spike-protein-based mutations, such as a change in protein structure, stability, neutralizing antibody interactions, RBD flexibility, and accessibility to hACE2, were analyzed. It is anticipated that the overall findings of our analyses will provide structural insights about different SARS-CoV-2 VoCs and the adaptive evolutionary tactics by which they maintain and increase their virulence in humans. We believe that the information provided in this review can help researchers predict the dynamism of SARS-CoV-2. It will be helpful for rationally designing the next generation of vaccines and therapeutics to deal with the COVID-19 pandemic.

## 2. Materials and Methods

The primary FASTA sequence of the spike glycoprotein (ID: P0DTC2) was extracted from the UniProt database (https://www.uniprot.org; accessed on 15 February 2023) and used as a reference sequence. The desired mutated sequence was used further as a query sequence for homology modeling. The tertiary structure of D614 (wild type) was taken from PDB ID: 6VYB. The mutated G614 was retrieved from PDB ID 6XS6, the crystal structure of the SARS-CoV-2 spike D614G variant. The structure of PDB ID 6VXX was used as a template for homology modeling of A222V, K417N, and Y369C mutations, since these mutations are found in the buried region of the spike glycoprotein. The closed conformation of spike glycoprotein would enforce more impact on the correct folding of buried residues. Additionally, E484K, the (PDB ID: 6VYB) template was selected, in which one of the RBD domains is located towards the top position. In this case, 6VYB was chosen due to the fact that E484 is found on the exposed surface region. It provides better modeling due to the use of open-state spike glycoprotein as a template. The PDB ID: 6M0J (RBD and hACE2) crystal structure was selected as a template for N501Y, L452R, Y453F, and T478K mutations, since these mutations affect hACE2 interaction. The PDB 6ZDG was selected to assess the EY6A antibody interaction with the spike glycoprotein. This 6ZDG is the crystal structure of the association of three complexes of disordered spike ectodomain with the bound EY6A Fab. The RBD hACE2 complex structure was retrieved from the PDB structure (PDB: 6M0J). The European Bioinformatics Institute’s Clustal Omega Server (www.ebi.ac.uk/Tools/msa/clustalo/; accessed on 22 February 2023) was used to perform template and target sequence alignment, and suitable chains (6VXX chain A, 6VYB chain A, 6XS6 chain A, and 6M0J chain B) were selected for the corresponding mutants to study the homology modeling.

### 2.1. Molecular Modeling of Spike Mutations

The corresponding PDB structure was used as a template (described above) to construct the mutated spike glycoprotein model, on the Modweb web server. The ProSA (protein structure analysis) web server was further consulted to detect the error in the three-dimensional structure of the mutated protein [17]. Procheck was used to assess the stereochemical properties of the protein [18]. Amongst the generated models, the one with the lowest root mean square deviation (RMSD) was selected for the analysis involving superimposition onto the corresponding template. The mutations were further energy minimized by the Chiron web server (www.dokhlab.med.psu.edu/Chiron; accessed on 24 February 2023) [19]. Different models were generated, and were further visualized by Pymol software (DeLano, W.L. The PyMOL molecular graphics system is available online: https://pymol.org/2/ (accessed on 15 February 2023).

### 2.2. Solvent Accessible Surface Area Calculation

Solvent accessible surface area (SASA) is a crucial factor in protein stability. This area was calculated by a hypothetical solvent sphere rolled over the van der Waals contact surface of the spike glycoprotein (PDB: 6VXX). Further, the SASA score suggests that amino acid residues of spike protein are buried inside or exposed on to the surface. Moreover, it indicates that the monomer’s amino acid is in contact with nearby monomer chains. The SASA score was calculated by Pymol 2.5 version software. In addition to the SASA and sequence profile, the β factor could also inform the differences between the interface regions, and the remaining protein surface was also considered [20].

## 3. Results

Mutations facilitate viral evasion of the host immune system, allowing the virus to survive in diverse environments, and can affect transmission and virulence, as has been reported with many viruses [21,22,23,24]. Several mutations in SARS-CoV-2 proteins have been reported, resulting in different VoCs [25,26]. Among the major structural proteins of SARS-CoV-2, the N protein resides in the ribonucleoprotein core, and the others are embedded on the surface of the viral capsid. The virus uses spike glycoprotein as a tool for the cellular receptor recognition via its RBD, and the neutralizing antibodies bind to its spike protein (especially RBD). This prevents its binding to host cell receptor, hence making it a viable antigenic target for vaccine development [27,28].

The S-protein-based mutation(s) with reference to the Wuhan strain caused major global concerns. The high frequency of mutation altered the binding with hACE2 interaction, and hence increased the rate of infection, raising the need to modify the neutralizing antibodies for better immunogenicity. The Covishield vaccine (ChAdOx1 nCoV-19) is approved in many countries, and showed a nine-fold reduction in neutralization activity in vitro against the Alpha variant compared to that seen with the non-Alpha lineage [8]. The vaccine-generated protection against the Alpha variant was reported at 90% in BNT162b2 (Pfizer-BioNTech, Mainz, Germany) [7], 70% in Ad26.COV2-S (Johnson & Johnson, New Brunswick, New Jersey, USA) [29], and 86% in NVX-CoV2373 (Novavax, Gaithersburg, MD, USA) [10]. The BNT162b2 (Pfizer-BioNTech) showed 75% vaccine-mediated protection against the Beta variant [7], and 88% efficacy against the Delta variant [30]. The vaccine-mediated protection against the Gamma variant reported for the Ad26.COV2-S (Johnson & Johnson) vaccine was around 68% [29].

D614G (aspartic acid to glycine at 614th position in spike protein) is now a worldwide established mutation and has been reportedly shown to increase the transmission of the SARS-CoV-2 [31]. Several other mutations were also found in SARS-CoV-2 at different times, giving rise to the evolution of different VoCs with increased transmission and virulence. The surface-exposed spike protein acts as a central component of SARS-CoV-2, and facilitates its entry into the host cell via interaction with the hACE2 receptor [32,33]. This has implications during vaccine development and immunotherapy. The spike protein facilitates host cell attachment and viral fusion with the host cell membrane [34]. The interaction of SARS-CoV-2 with host cells results in a conformational switching in the spike RBD (down to up conformation). The spike protein can access and establish the interaction with hACE2 in RBD up conformation, whereas the receptor (hACE2) is not accessible in RBD down conformation [32]. The receptor inaccessible state consists of all RBDs in the down position, whereas in the accessible state at least one RBD should be up. It is reportedly evident that spike RBD is the primary part of the spike protein that interacts with the host cell. Therefore, we deliberately selected for our analyses those spike RBD mutations (N439K, N501Y, Y453F, E484K, K417N, T478K, and Y369C) that are present in the majority of VoCs. Additionally, we selected another vital mutation (A222V) that is not present in the RBD but has a high entropic score. D614G was considered as a reference mutation due to its universal presence [35].

### 3.1. D614G Mutation

**Figure 2:**
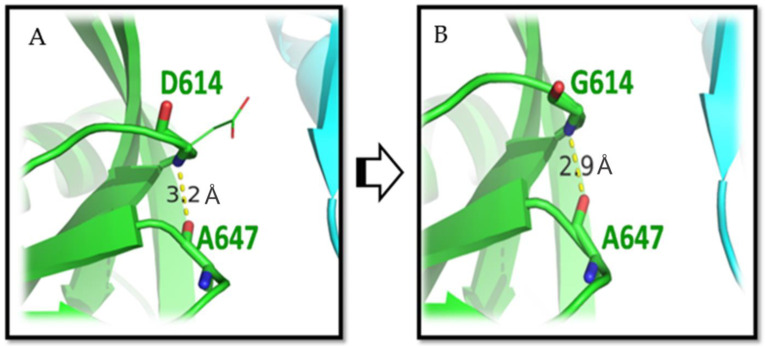
The D614G mutation in the SARS-CoV-2 spike protein. (**A**) D614 in the wild-type spike glycoprotein, and (**B**) G614 the mutated form of D614. The three-dimensional structure of the spike glycoprotein is represented colored by the chain. The yellow dotted line represents the hydrogen bond formation between residues 614 and 647, which shortens when aspartic acid mutates to glycine. The length between residues is shown in units of Angstrom (Å).

The D614G variant is a widespread mutation in which aspartic acid has been replaced with glycine at the 614th position of spike protein [36]. The tertiary structures of D614 (wild type) and G614 (mutant) were taken from PDB ID 6VYB and 6XS6, respectively. The D614 was found in the partially conserved region of the protein that interacts with T859 of the nearest spike protomer [37]. D614 also binds with A647 within the same protomer with main chain involvement, which could help to maintain the structural integrity. The interaction proximity of D614 with A647 is 3.2 Å, which decreases to 2.9 Å upon replacement of aspartic acid with glycine at the 614th position. Therefore, it potentially increases the robustness of the interaction (Figure 2). Thus, after mutation, G614 might interact with A647 more profoundly, and could change the orientation in the loop region of the same protomer as shown by our analyses. Subsequently, the hydrogen bond formed between D614 and T859 in the wild type (protein) is lost after the G614 mutation, and the protomer becomes more flexible and could gain access to the host receptor. Eventually, this leads to the higher infectivity of G614 variants than D614 [37]. Further, it is reported that the viral load is reduced, measured as the decreased Ct value in the RT-PCR. However, Ct values were seen to increase in the mutated G614 variants [38]. This possibility is also substantiated by findings wherein G614-variant infected hamsters produced elevated infectious titer compared with the wild type (D614).

These findings are in agreement with the others showing the increased viral load from the mutation in patients with COVID-19 and hence increased rate of transmission [37]. Therefore, it is presumed that the G614 mutation might have occurred under positive selection pressure [38], resulting in increased spike-protein stability and viral infectivity [39,40]. One study reported reduced (1.7- to 2.4-fold) SARS-CoV-2 neutralization by the BNT162b2 vaccine formulation against G614. The vaccine efficacy of BNT162b2 was observed to be 95% against the circulating virus (G614 mutation) under clinical trials [41]. Therefore, this mutation should be used for the design of the vaccine as it is the result of fitness average rather than genetic drift or/and founder effects [37].

### 3.2. A222V Mutation

**Figure 3:**
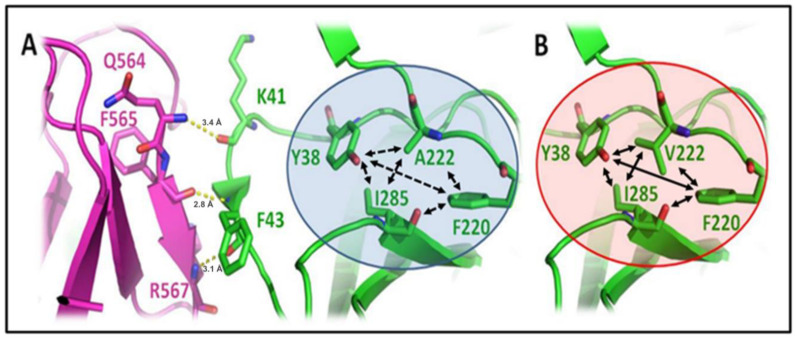
The wild-type A222 in the spike glycoprotein mutates to V222, shown in the A222V mutation. The green illustration represents the NTD, with the residues residing close to the RBD in magenta. (**A**) Neighboring hydrophobic residues surround the wild-type A222 in the spike glycoprotein: Y38, I285, and F220. The representation is shown in the blue circle; (**B**) The mutated V222 in the S protein is surrounded by hydrophobic residues; Y38, I285, and F220 make a comparatively strong hydrophobic core (shown in the red circle). The dotted/straight (double-headed arrow) illustrates the hydrophobic interaction. The length between residues is shown in units of Angstrom (Å).

A222V is a widespread mutation wherein alanine residue is replaced by the hydrophobic amino acid valine at the 222nd position in the spike protein. The A222V mutation was found in the N terminal domain (NTD). It has the second highest frequency, following D614G in the B.1.177 lineage, and was widely spread in Europe [42]. There has been a lack of evidence for the impact of A222V mutation on viral transmissibility and infectivity, since this viral mutation was likely to be accidental rather than the result of selection pressure [26]. A222V mutation could mediate protein stability and its conformation. Hence, this mutation and possible outcomes thereof are discussed in the present paper. The A222 is surrounded by other hydrophobic residues (Tyr38, Phe220, and Ile285) in the NTD of spike protein (Figure 3A; blue circle). Since valine is more hydrophobic than alanine, it is considered that the motif after mutation becomes more hydrophobic than the wild type strain, as shown in Figure 3B (red circle). The weaker hydrophobic interaction is indicated by the dotted line (under the blue circle) while the stronger hydrophobic interaction is shown by the straight line (under the red circle). The NTD of one spike protomer is in proximity with the nearby RBD (333 to 528 amino acids) of another protomer (shown in magenta color; Figure 3). Hence, the mutation may affect spike RBD flexibility via long-range effects of hydrophobic residues (Val222, Tyr38, Phe220, and Ile285) at NTD. The wild-type interaction was observed between the K41 and F43 of NTD residue with Q564, F565, and R567 of RBD. Following mutation, K41 and F43 orientation may move away from Q564, F565, and R567, and subsequent interaction could be weaker, affecting the hinge binding of the RBD. As a consequence, the RBD becomes more accessible enabling binding with hACE2.

### 3.3. S477N Mutation

**Figure 4:**
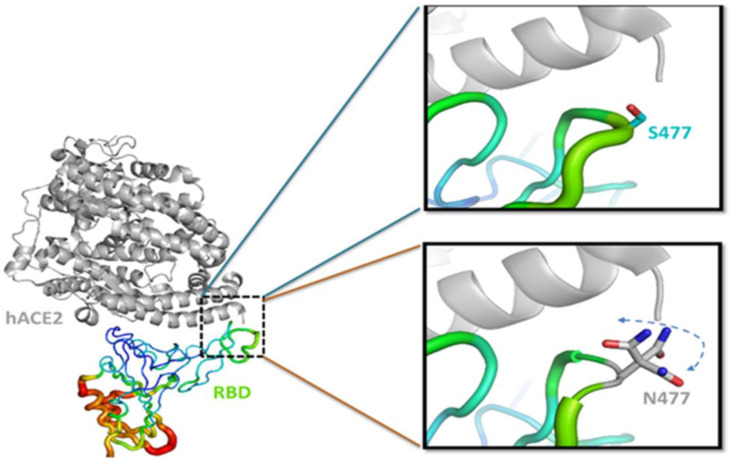
The RBD and hACE2 interactions were taken from the PDB structure (PDB: 6M0J). The hACE2 is shown in gray and RBD as a β factor diagram (shown in rainbow). The S477 (wild type) is magnified in the upper right box. The N477 (mutated) is magnified in the lower right box. The side chains of N477 are in different rotamers, increasing its propensity of binding with hACE2 in comparison with the wild type.

In the S477N variant, serine is mutated to asparagine at the 477th amino acid position of the spike. The mutation was found in the receptor binding motif (RBM) of RBD. RBM is the actual interface of the spike RBD that is engaged in the interaction with host receptor ACE2 [43]. This mutation occurred in the highly flexible region of the protein, as evaluated by normal mode analysis. The residues starting from S477 to G485 of the spike are probably involved in interactions with the N-terminal helix of hACE2, as measured by RMSF (root mean square fluctuation) under computational simulation analysis [43]. We also evaluated the β factor in RBD-hACE2 interaction (PDB:6M0J) using Pymol software. The results depict that the residues near to S477 are highly flexible and, after mutation to N477, might have a higher probability of binding with nearby amino acid residues from hACE2. These interactions might be possible because of an increase in the side-chain and rotamer orientations of N477 in contrast with S477 (Figure 4). The S477 residue found in the solvent-exposed loop region is also recognized by the C102 antibody [42].

Therefore, it is considered one of the most important mutations and could be involved in viral tropism [44]. Overall data suggest that S477N mutation strengthens the binding of the spike with the hACE2 receptor and interferes with the binding of neutralizing antibodies [26,43]. Thus, S477N mutation plays a significant role in the interaction with hACE2 and confers resistance to convalescent sera and antibodies, subsequently increasing infectivity [45,46]. Many other mutations were also reported from various countries. The most prominent mutations were reported from the UK, South Africa, Brazil, Spain, and India. These mutations produce a different category of variants, as discussed below.

### 3.4. Alpha Variant

Alpha variants are also categorized as B.1.1.7 lineage. Viruses of such lineage are predicted to be more transmissible than the wild-type SARS-CoV-2 strain, which was reported in November 2020 in the United Kingdom [47]. The significant mutations reported in Alpha variants are N501Y, N439K, and Y453F, and are explained below.

#### 3.4.1. N501Y Mutation

**Figure 5:**
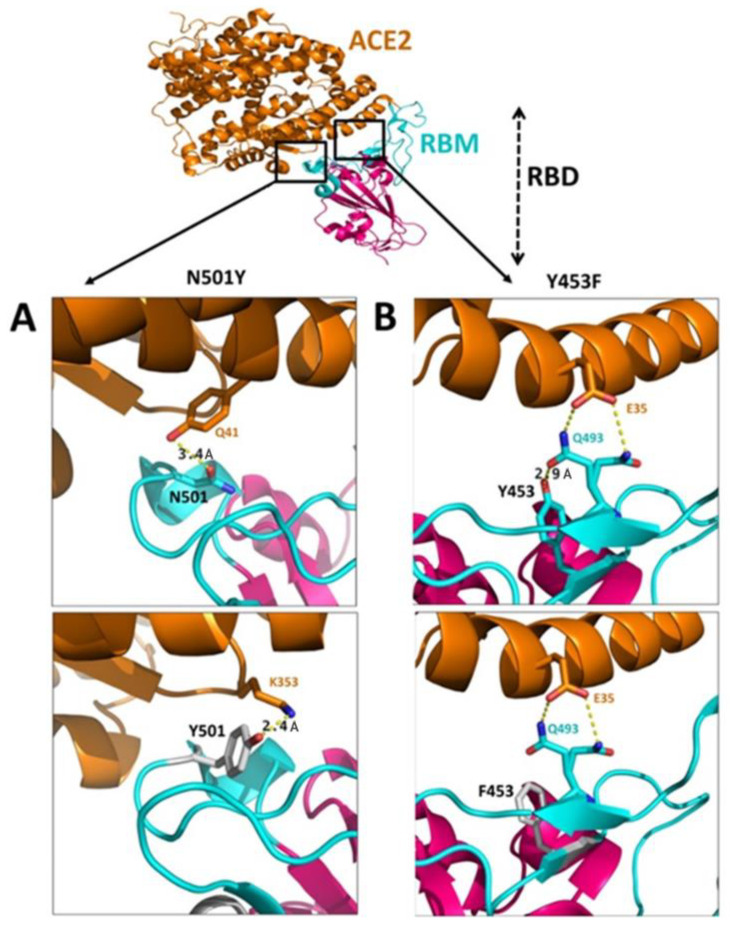
The interaction between RBD and hACE2 (adapted from the PDB structure (PDB: 6M0J)). The hACE2 is in orange, RBM is in cyan, and the RBD (except RBM) is in magenta. (**A**) The N501Y mutation: the wild-type N501 of RBM interacts with Q41 of hACE2 (3.4 Å), which after mutation (Y501) binds more robustly with K353 of hACE2 (2.4 Å); (**B**) The Y453F mutation: the wild type Y453 interacts with Q493 of RBM (2.9 Å) and subsequently restrains Q493 (reside in two rotamers) interaction with E35 of hACE2. The mutated F453 loses the binding with Q493, subsequently increasing the propensity of Q493 (reside in two rotamers) interaction with E35 of hACE2. The upper panel represents the wild type, whereas the lower panel denotes the mutated interaction. The wild types N501 and Y453 are in cyan, and mutated Y501 and F453 are in gray. The length between residues is shown in units of Angstrom (Å).

In the N501Y mutation, the asparagine changed to tyrosine at the 501st amino acid position of spike RBD [48]. The wild type N501 interacted with glutamine (Q41) of hACE2 via hydrogen bond with the interaction proximity of 3.4 Å. Following the Y501 mutation, the interacting interface changed to K353 with a proximity of 2.4 Å (Figure 5A) and could result in an increased binding affinity of the spike with hACE2, as demonstrated by the in silico analysis [49] and fluorescence-activated cell sorter (FACS)-based bioassay [50]. The N501Y mutation is associated with other mutations, namely, N439K, H69/V70 deletion [51], and therefore considered a critical mutation. Altogether, N501Y mutation in the RBD appears to play a critical role in the transmission and virulence of SARS-CoV-2 [52]. Although the N501Y mutation has been shown to increase the affinity with hACE2, reduction in the neutralizing activity is not seen with the Pfizer-BioNTech vaccine [53].

#### 3.4.2. Y453F Mutation

In the Y453F mutation, tyrosine positioned at the 453rd amino acid position of spike RBD was replaced by phenylalanine (Y453F). The tyrosine and phenylalanine are similar in structure except for the presence of a hydroxyl group in tyrosine at para position, which does now allow mutation to impact the native state of the RBD. This change enhances the binding affinity between RBD and hACE2 and makes it a crucial mutation. The wild-typeY453 interacts with Q493 of the RBD (which resides in two different rotamers) in the same trimer (Figure 5B). After the Y453F mutation, the interaction between tyrosine (453) and glutamine (493) is lost. Consequently, Q493 is accessible to the interaction with E35 of hACE2 with prominence (Figure 5B). However, Y453F mutation reportedly decreases the interaction between neutralizing antibodies (CV07-250, COVA2-04, CC12.1, and CC12.3) and spike protein, compared with the wild type [54,55]. Moreover, the Y453F mutation in the SARS-CoV-2 mink variant displays a pronounced increase in ACE2 affinity but does not challenge antibody neutralization. Further, Y453F mutation harboring SARS-CoV-2 has been shown to escape neutralization with RGN10933 mAb(s) [56].

#### 3.4.3. N439K Mutation

The polar uncharged asparagine is present at the 439th position of spike RBD. Asparagine has been replaced by a positively charged lysine (N439K) in this mutation. This imposes structural changes in the RBM, and hence a new salt bridge interaction with E329 of hACE2 is established [57]. The establishment of the salt bridge interaction helped neighboring residues to assist in increasing the robustness in the RBM-hACE2 bonding. Furthermore, N439K variants showed resistance to the REGN10987 [57,58] and DH1047 neutralizing antibodies [59]. These findings support our interpretation that the N439K variant could potentially lead to viral escape from neutralizing by antibodies. Overall, it can be extrapolated from analyses that spike RBD-based mutations (e.g., N439K, Y453F, and N501Y) could enhance the interaction of SARS-CoV-2 with hACE2 and could result in escape from neutralizing antibodies, viral transmission, and infectivity.

### 3.5. Beta Variant

The Beta variant is categorized as B.1.351 lineage, and is more transmissible according to reports from South Africa [60,61]. The significant mutations in Beta variants are N501Y, E484K, and K417N [61]. The N501Y mutation has been already discussed in relation to the Alpha variant.

#### 3.5.1. E484K Mutation

**Figure 6:**
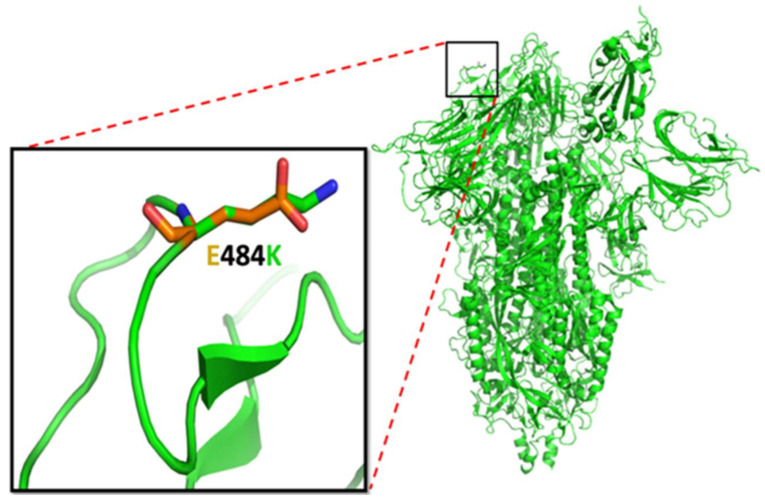
The E484K mutation: the structure in green represents the spike glycoprotein. The magnified black box represents the surface-exposed E484K mutation. The glutamic acid and mutated lysine residue were superimposed on the same region (shown in the box). The orange color denotes the negative charge of glutamic acid, and the green color depicts the positive charge of lysine. The mutated lysine changes the surface property because of its positive charge, and length of side chain compared with glutamic acid.

Glutamic acid replacement by the lysine gave rise to the E484K mutants. The E484 site has recently been investigated by many research groups their results indicate the reduced neutralization of SARS-CoV-2 by neutralizing antibodies in the sera [62,63,64,65]. The interaction between RBD and hACE2 in E484K mutation of spike protein was investigated by molecular dynamics simulation using the molecular mechanics-generated born surface area (MMGBSA) method. The results showed an increase in the binding affinity between RBD-hACE2 [66]. The charge substitution (change of negatively charged glutamic acid to positively charged lysine) of the E484K mutation impacts adaptive viral evolution that leads to higher virulence (Figure 6) [67]. The latter could be manifest in E484K mutants due to more robust interaction between RBD-hACE2 and reduced antibody-led neutralization [63,68].

#### 3.5.2. K417N Mutation

**Figure 7:**
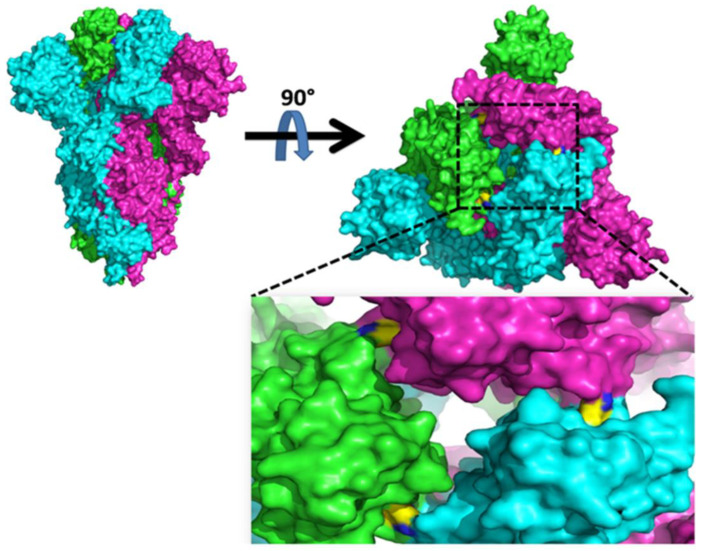
The spike glycoprotein in the trimeric state is shown as surface representation in the (**upper left**). The protomer is shown in green, cyan, and magenta. The top view shows K417 interaction with N370 (between nearby protomers) shown as a yellow surface (**upper right**). The black dotted box zooms out to show the K417 and N370 interaction (**below right**).

First reported in South Africa, the K417N mutation located in spike RBD involves lysine being substituted with asparagine at the 417th position. This mutation was also reported in India along with E484Q and L452R, and the variant termed Delta Plus by WHO [69]. The K417 is a very specific amino acid in spike RBD that interacts with N370 of nearby spike protomer in its down state (Figure 7, shown in yellow). Hence, in K417N mutation harboring variants, there is a possibility that more than one RBD can be opened up and remain available for interaction with hACE2, unlike that seen with the wild-type virus [70]. The overall impact of this mutation could be an increase in viral infectivity [71]. Essential site scanning analysis (ESSA) revealed that K417N serves as an allosteric modulating binding site regulating the conformational dynamics of the RBD-hACE2 complex [72]. K417T mutation was reportedly also found in the Gamma variant where tyrosine replaced the asparagine [73]. Thus, Beta and Gamma variants possess alternative substitutions at the 417th amino acid in the spike, but the antigenic effects decrease the hACE2 binding affinity with both the mutations [73,74].

### 3.6. Gamma Variant

The Gamma variant is categorized as P.1 lineage, and was first detected in Brazil [75]. The major mutations in Gamma variants are N501Y, E484K, and K417T, and these mutations have been described in the previous section.

### 3.7. Delta Variant

The Delta variant is the B.1.617.2 lineage, and is considered the most widely circulating variant of SARS-CoV-2 [76]. Major mutations in the RBD in the Delta variant include L452R and T478K [77].

#### 3.7.1. L452R Mutation

**Figure 8:**
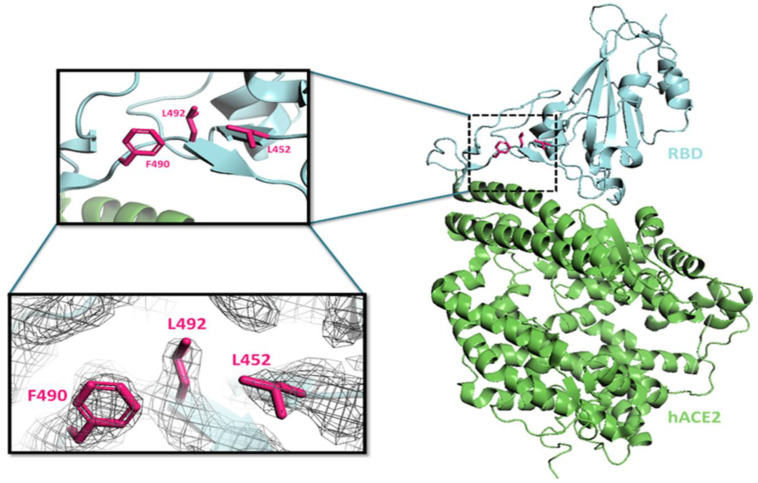
The RBD and hACE2 interactions were taken from the PDB structure (PDB: 6M0J), shown on the right. The structure of hACE2 is shown in green and RBD in cyan. The black dotted box is magnified to represent the L452, L492, and F490 residue in the RBD and is shown in magenta in the upper left box. The electron-density map for these residues (L452, L492, and F490) as shown in the below left box.

In this mutation, the leucine is replaced by arginine at the 452nd amino acid position in the spike RBD. The L452 residue interacts with L492 and F490 residues within the same protomer via hydrophobic interaction in the wild type (Figure 8). The single and double variants of Delta form harboring L452R mutations in the spike protein were reported during the second wave of COVID-19 in India [78]. Due to the L452R mutation, the hydrophobic patch on RBD was eliminated, and subsequently, the binding energy with hACE2 was seen to decrease [79]. The residues L452 and E484 are involved in direct contact with the heavy chain (I103 and V105) of mAb P2B-2F6 [79,80]. Therefore, this mutation (L452R) might result in breaking down the interaction with I103 and V105 residues and could assist the virus in antibody escape. The L452R mutation independently spread in many lineages all across the globe from December 2020 to February 2021. This indicates that this mutation could have resulted due to the viral adaptation [26,81].

#### 3.7.2. T478K Mutation

**Figure 9:**
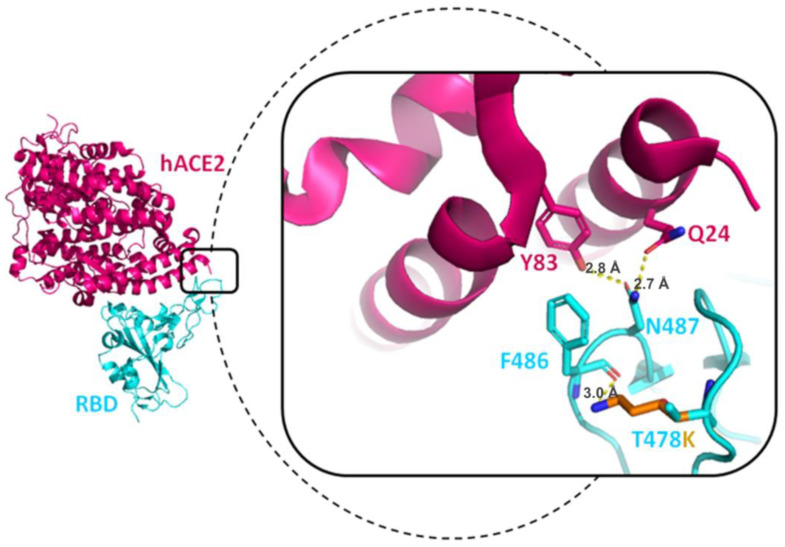
The RBD and hACE2 interactions were taken from the PDB structure (PDB: 6m0j), shown on the left. The structure of hACE2 is shown in magenta and RBD in green. The dotted line is magnified to represent the result of the T478K mutation. The length between residues is shown in units of Angstrom (Å).

The T478K mutation spread predominantly in North America, especially in Mexico [82]; it is another mutation found on the spike/hACE2 interaction interface. The structure was elucidated by using PDB: 6M0J. The T478 in the wild type state does not bind with any nearby residue, as shown in Figure 9. The non-charged threonine at the 478th position mutates to a long positively charged side chain of lysine (T478K), subsequently altering the electrostatic surface of the spike protein. The mutated lysine at the 478th residue may bind with F486 of RBD and support the loop stability of RBD. The N487 of RBD already involved with hACE2 interaction (Y83 and Q24) becomes more robust and stable after the T478K mutation. The lysine side chain may increase steric hindrance and could possibly bind with nearby hACE2 residues. This specific Delta variant is found within the epitope region of the neutralizing antibody class 1 category [77], making the virus more infectious and transmissible [83].

### 3.8. Delta Plus Variant

The Delta Plus variants are named as B.1.617.2.1 lineage and are similar to the Delta variants [84]. They carry the K417N mutation along with existing mutations in the Delta variant. The Delta Plus variants (L452R, E484Q, K417N) have shown greater affinity towards lung mucosal lining than seen with the other variants [85]. They are resistant to mAbs (Casirivimab and Imdevimab) that are used for the treatment of COVID-19 [86,87]. The Indian SARS-CoV-2 Consortium on Genomics (INSACOG) has recently reported the increased transmissibility of Delta Plus variants [88].

### 3.9. Omicron Variant

**Figure 10:**
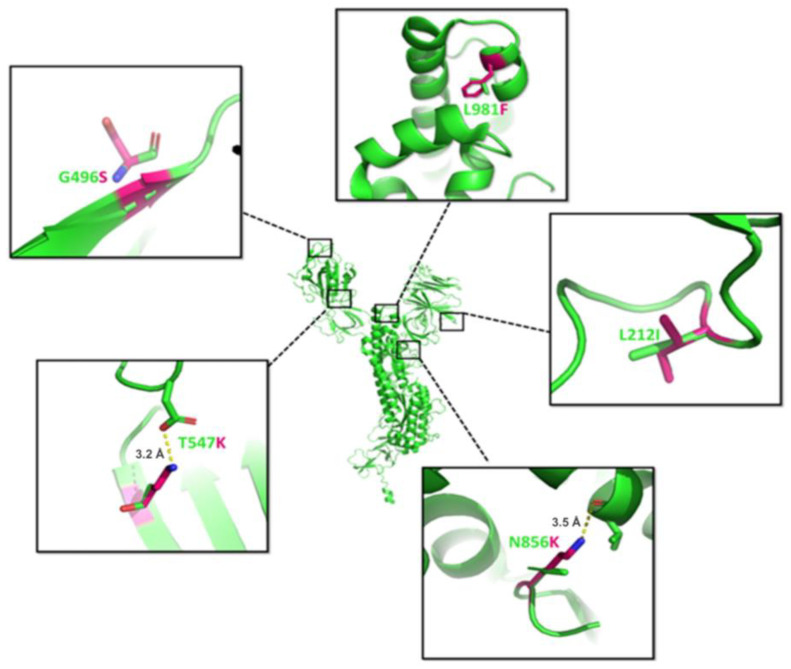
The structure of the spike (PDB: 6VXX) is shown in green (center). The dotted line is magnified to represent the results of L981F, L212I, N856K, T547K, and G496S mutations, where the mutated amino acid is shown in magenta and wild-type amino acids as green sticks. The length between residues is shown in units of Angstrom (Å).

The Omicron variant (B.1.1.529) is considered the most recent VoCs (considering 35 mutations) with increased risk of reinfection [89]. The RBD mutations of Omicron are G339D, S371L, S373P, S375F, K417N, N440K, T478K, E484A, Q493R, Q498R, S501Y, and Y505H [90,91]. The Omicron variants also have other unique deletions/mutations such as ΔN211, L981F, L212I, N856K, T547K, G446S, and G496S (https://covariants.org/variants/21K.Omicron; accessed on 1 March 2023) [91]. From among these mutations, the L981F, L212I, N856K, T547K, and G496S are shown in Figure 10. The most strategical approach used in the COVID vaccine was based on proline dimer mutation at K986 and V987, which has been shown to increase spike protein stability [28]. However, the proline mutation at 986 (K986P) may break the salt bridge interaction established between protomers that confers the strength to the trimer [92]. Hence, L981F mutation was introduced by cavity-filling substitution between the two helices to maintain K986 as the wild type in the Omicron variants, which showed similar effects to the proline dimer. This is the most crucial mutation discovered in the BA.1 lineage and is absent in the BA.2 lineage of Omicron (https://covariants.org/shared-mutations). Thus, this single mutation potentially increases the stability of the spike, which in combination with higher polar RBD mutation makes the Omicron BA.1 linage far more infectious [93]. BNT162b2-vaccinated individuals showed lower neutralization (~27 fold) against Omicron than against the D614G mutation, demonstrating its escape from vaccine [94]. The L212I mutation occurs in the NTD region where leucine replaces more hydrophobic isoleucine, which could change the binding activity and affect the stability of the spike protein [95,96]. N856K is another mutation that substitutes lysine and increases the polar side chain, which binds with the main chain of valine (V963) [97]. The T547K mutation also shows a similar pattern, wherein substituted lysine binds with aspartic acid (D389). The glycine at the end of the helix mutates to serine in the G496S mutation and thus increases the stability between the helix and loop region. Thus, G496S mutation increases the RBD stability of spike protein. Collectively, all the mutations under the Omicron variant increase the ability to bind with the hACE2 receptor as well as viral transmission [98].

#### Y369C Mutation

**Figure 11:**
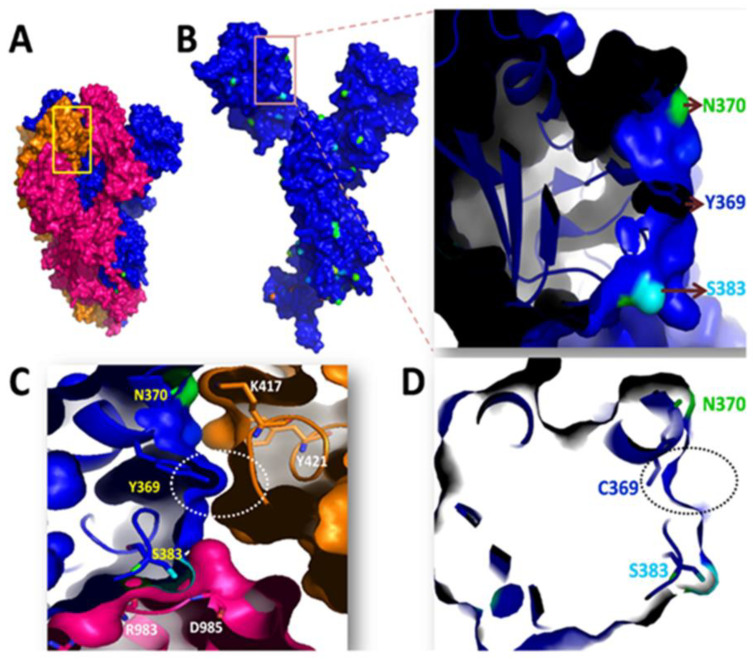
(**A**) The spike glycoprotein state is illustrated in the trimeric state, with protomers depicted in magenta, blue, and orange; (**B**) The surface area accessibility of spike protein is represented in one of the protomer states (blue). The inset represents the magnified view of Y369 residue along with nearby exposed amino acids (N370 and S383). The N370 is described in green, and S383 in cyan; (**C**) The involvement of Y369 residue in cavity filling is illustrated for different protomers (shown in a white circle) for structural integrity. The Y369, N370, and S383 of one protomer (blue) are shown in yellow; K417, Y421, R983, and D985 of nearby protomers (orange and magenta) are shown in white; (**D**) The mutated C369 lacks the binding cavity as shown in the black circle.

In the Y369C mutation, tyrosine at the 369th amino acid position of spike RBD is changed to cysteine. Y369C-harboring SARS-CoV-2 spike variants have previously been reported [99]. The surface area accessibility of the spike was calculated, showing that Y369 is involved in the spike monomer’s interface region. We found that N370, S383, Y369, K417, and Y421 amino acids are present in an interface region of the spike with higher solvent accessible surface area (SASA) scores (Figure 11A and Table S1). Among the aforementioned amino acids, K417 mutation has already been found in different variants. This suggests that amino acids present in the interface region of the spike are mutation-prone and thus immensely relevant to the virulence. Since Y369 is also present in the interface region, the mutant Y369C might have the same impact as K417N and could lead to the emergence of a newer variant. Though tyrosine is hydrophobic, the phenolic hydroxyl group of tyrosine is more acidic than aliphatic hydroxyl amino acids (e.g., serine or threonine). This makes tyrosine more involved in the interaction, leading to better binding. Y369 was found close to N370 and S383 (Figure 11B). N370 of chain A interacts strongly with K417 and Y421 of chain C, whereas S383 interacts with R983 and D985 of chain B (Figure 11C). This interaction was facilitated by filling the cavity with Y369 between chain A and chain C (Figure 11 C; white circle). Therefore, Y369C mutation removes the cavity (Figure 11D; black circle) and results in structural changes that weaken the interactions of N370 with K417/Y421 and S383 with R983/D985. The weakening of the interactions might maintain the RBD in an open state, easily able to bind with the hACE2 receptor. K417 has already been reported as an essential mutation, hence it could be presumed that the Y369C variant might dominate in the near future.

**Figure 12:**
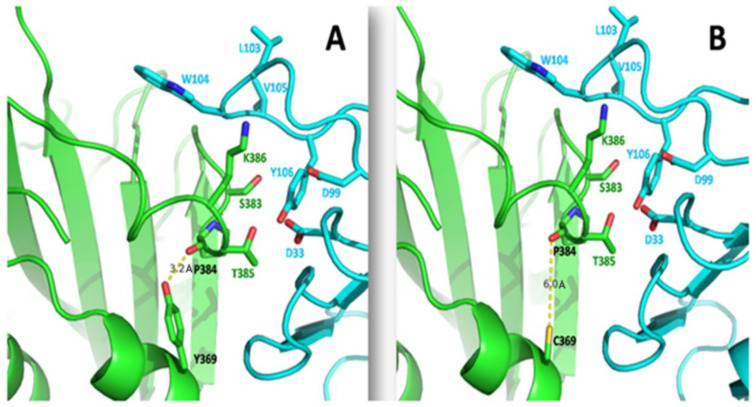
The interactions among the EY6A antibody with spike RBD region were taken from PDB: 6ZDG. The structure in green represents the RBD region of spike glycoprotein. The structure in cyan represents the EY6A Fab. (**A**) The interaction between Y369 and P384 is indicated, with a bond distance of 3.2 Å; (**B**) The interaction of mutated Y369C with P384 is shown, where the interaction distance increases to 6.0 Å which subsequently increases the flexibility of the binding region of spike glycoprotein. The yellow dotted line represents the hydrogen bond interaction. The length between residues is shown in units of Angstrom (Å).

Furthermore, Y369 interacts with EY6A, an antibody isolated from COVID-19 patients, highlighting this mutation as a matter of concern since it could lead to antibody-escape mutants. The crystal structure reveals that EY6A Fab binds with the RBD of the spike reported in the PDB structure (PDB: 6ZDG). This interaction is seen between S383, T385, and K386 of the RBD and D33, D99, L103, W104, V105, and Y106 of the heavy chain of Fab. Y369 plays an essential role in maintaining RBD mAb interaction effectively intact; the Y369 of RBD is considered one of the important amino acids, binding with P384 (3.2 Å) to stabilize the loop-prone S383, T385, and K386 of the RBD domain (Figure 12A). After mutation to C369, its interaction with P384 (6.0 Å) loosens (Figure 12B). The rigidity of S383, T385, and K386 amino acids of the RBD is lost due to the Y369C mutation and increased RBD flexibility. Further, a flexible RBD binds with hACE2 receptors more easily and hence increases the transmission of the virus.

## 4. Discussion

The in-depth elucidation and understanding of adaptive evolution in novel coronavirus SARS-CoV-2 is critical for designing the vaccines and therapeutic strategies to contain and/or eradicate the current COVID-19 pandemic [100]. Different kinds of natural mutations occur in viruses to acclimatize them to diverse environments, withstand selection pressure, and maintain their existence and adaptive evolution for survival and virulence. Similarly, the novel coronavirus SARS-CoV-2 also mutates different parts of its genome, encoding for structural and non-structural proteins and antigenic determinants such as the spike protein. Spike protein, the major antigen of SARS-CoV-2, is targeted by the currently licensed vaccines against COVID-19 [101]. Several variants of SARS-CoV-2 from all across the globe have been reported in the recent past, harboring mutations in the gene encoding for spike protein antigen [102]. Many of these variants are considered VoCs due to their increased transmissibility, infectivity, and virulence [103]. The major VoCs, including Alpha (B.1.1.7), Beta (B.1.351), Gamma (B.1.1.28.1), and Delta (B.1.617.2) pose concern from a public health perspective since they are associated with higher infectivity, transmission rate, and potential immune evasion mechanisms [24,104,105]. The present study illustrates the structural changes using different bioinformatics tools to understand the possible impact of these mutations on spike protein stability, its accessibility to ACE2 receptor, RBD flexibility, neutralizing of antibody interactions, antigenicity, antigenic diversity, and their potential implications on viral virulence.

Our study has primarily focused the spike RBD-based mutations, because the spike is the prime interacting interface with the host ACE2 receptor and is part of most vaccines (licensed and under clinical development). The experimental evidence corroborates our in silico analyses. For example, the D614G mutation was reported to help increase viral infectivity [36,38,40], transmission [39], and viral replication in the upper respiratory tract (URT) [37]. The A222V mutation resides in the NTD, and hence does not involve receptor binding but might affect spike glycoprotein conformation. These conformational changes in turn assist the viral entry into the host cell [42]. One mutation in the Alpha variant (N501Y) apparently plays a critical role in viral transmission, whereas other mutations such as N439K and Y453F could potentially help the virus escape from being neutralized by the antibodies. Recently, Wang et al. examined the neutralization potential of plasma isolated from mRNA-vaccine-immunized individuals using pseudo virus containing N501Y, E484K, and K417N mutations [106] and showed the slightly reduced neutralizing activity of vaccinated sera against this variant. Furthermore, neutralization of this variant by mAb(s) (isolated during the experiment) was also affected. A small difference in vaccine effectiveness was seen with the Delta variant compared to that seen with the Alpha variant following two doses of COVID-19 vaccine [30]. Pouwels et al. investigated different licensed COVID-19 vaccines (BNT162b2, ChAdOx1, and mRNA-1273) in a community-based survey of arbitrarily selected households within the United Kingdom. They reported that all the vaccines were found to be efficacious, and vaccination reduced new infections, but the effectiveness was found to be reduced with Delta variants [107]. Planas et al. reported that antibodies elicited by the Pfizer and AstraZeneca vaccines were observed effective against the Delta variant. However, they were about three- to five-fold less potent when activity against the Alpha variant was compared, as validated by the in vitro neutralization assay [77]. Recently, Yadav et al. assessed the neutralization potential of convalescence sera obtained from COVID-19-recovered subjects and Covaxin (BBV152, inactivated SARS-CoV-2 vaccine)-vaccinated individuals against Beta and Delta variants. The Covaxin conferred significant protection against both the variants. However, slight reductions in neutralization against the Beta and Delta variants was observed in the in vitro assay. It is imperative to conduct vaccine effectiveness studies based on field trials to understand the actual impact of the reduced in vitro neutralization activity on the efficacy of vaccines that are in use in different countries [108].

## 5. Conclusions

The research findings suggest that epitopes are relatively immunogenic, conservative, nontoxic, capable of inducing cytokine production, and at high probability of becoming exposed to the spike glycoprotein of SARS-CoV-2. Hence, our analysis results emphasize that major surface-exposed spike glycoprotein may be a direct or indirect target for vaccine design and development. The present study showed the selection of highly virulent mutants A222V, N439K, N501Y, L452R, Y453F, E484K, K417N, T478K, L981F, L212I, N856K, T547K, G496S, and Y369C. Immune evasion and possible antagonism between the innate immune system and the Alpha and Delta variants are associated with increased transmissibility and severity of the disease. Moreover, due to their ability to evade humoral immunity and raising the possibility of reinfection, the Beta and Gamma variants are associated with increased transmissibility. Therefore, our major concern has been to cover the prime antigenic region of the whole spike glycoprotein in SARS-CoV-2 variants, to assist in vaccine design for all COVID-19 strains.

Manifest effects of the reduced neutralization activities on vaccine effectiveness, disease outcome, severity, and infection are not yet clear. Therefore, our analysis highlights the need for comprehensive population-based surveillance and monitoring of the effectiveness of different vaccine formulations. All spike-based vaccines licensed for the immunization across the globe are effective in preventing SARS-CoV-2 infection. However, different mutations have been reported with varying degrees of impact in terms of reduced neutralization of serum samples against different variants in assays in vitro. Notably, most of these published vaccine efficacy reports correlate against VoCs and depend upon in vitro pseudovirus neutralization, and may not be completely translated into actual efficacy. Based on the above analyses, it is difficult to draw a conclusion the actual manifestation of these mutations and their impact on vaccine efficacy against various VoCs, due to the availability of limited experimental and field-trial data. Thus, in-depth understanding of the mutation dynamics in the SARS-CoV-2 antigenic component is crucial when designing the next generation of vaccines and monoclonal antibodies for addressing COVID-19. Overall, our analyses can help researchers to design novel vaccines with updated information about potent mutants. Furthermore, the report provides an update on the adaptive evolution of the virus, aiming at developing resilience against the vaccine. Population-level data about vaccine breakthrough infection concerning individuals’ immune competence and cell-mediated immunity should be considered, in order to develop further understanding of the effectiveness of COVID vaccines. Collectively, the findings of our analysis could be helpful in rationally designing the new generation of vaccines and biotherapeutic candidates against the evolving circulating strains of SARS-CoV-2 variants.

## Data Availability

All figures and tables has been given in the manuscript and no other data is left to be disclosed.

## Supplementary Materials

The following supporting information can be downloaded at: https://www.mdpi.com/article/10.3390/v15040856/s1, Table S1: Surface-exposed amino acid monitored by beta factor as shown in green color.

## Abbreviations

hACE2	Human angiotensin converting enzyme 2
mAbs	Monoclonal antibodies
NTD	N terminal domain
RBD	Receptor binding domain
RBM	Receptor binding motif
RMSD	Root mean square deviation
SARS-CoV-2	Severe acute respiratory syndrome coronavirus 2
SASA	Solvent accessible surface area
VoC	Variant(s) of concern
WHO	World Health Organization
