# Supplementary material for: Understanding Mutations in Human SARS-CoV-2 Spike Glycoprotein: A Systematic Review & Meta-Analysis
Source: Viruses. 2023 Mar 27;15(4):856. doi: 10.3390/v15040856 (PMC10142771; doi:10.3390/v15040856)
Supplement: Supplementary file 1 [file viruses-15-00856-s001.zip › viruses-2297720-supplementary.pdf]

Supplementary Table S1. Surface exposed amino acid monitored by Beta factor as shown in green in color.

GLN	A	965	206.9	223.59	213.8	15.84	2.49
GLN	A	913	212.628	221.002	147.701	16.99	2.24
PRO	A	1090	200.141	208.24	145.755	14.49	2.16
SER	A	1123	198.928	198.067	142.895	16.54	2.01
ILE	A	788	230.951	227.055	171.865	7.92	1.35
ASN	A	914	205.452	225.325	145.675	17.09	1.33
ALA	A	701	183.307	222.065	171.734	15.76	1.29
GLU	A	702	176.789	218.647	170.545	16.22	1.29
SER	A	758	223.878	207.111	211.394	16.99	1.29
TYR	A	904	219.875	222.057	152.877	17.33	1.29
ASN	A	703	184.728	222.351	165.388	18.09	1.27
ASP	A	796	220.654	236.008	156.463	15.26	1.24
TYR	A	707	184.367	207.847	156.949	17.6	1.21
SER	A	758	224.478	208.452	213.933	8.18	1.16
PHE	A	43	209.724	243.035	221.619	8.9	1.15
LYS	A	790	233.473	235.937	168.221	18.91	1.15
VAL	A	1094	195.011	213.091	149.982	23.06	1.14
TYR	A	200	202.35	237.532	240.866	17.7	1.09
GLN	A	1002	207.824	213.128	214.699	18.71	1.09
SER	A	383	193.393	208.265	239.378	8.33	1.08
ARG	A	983	217.832	226.012	236.813	16.28	1.05
PHE	A	565	181.124	195.504	223.28	16.13	1.02
ASP	A	568	183.682	202.019	213.585	17.08	1.01
GLY	A	669	182.615	218.177	189.272	9.1	0.95
LYS	A	854	217.015	238.134	211.144	19.69	0.95
GLN	A	895	230.077	227.463	155.12	18.66	0.95
GLN	A	1005	213.652	211.602	212.126	16.59	0.93
ASN	A	370	189.208	213.361	254.718	15.55	0.91
LEU	A	864	228.978	227.668	188.24	16.54	0.91
ALA	A	668	183.568	217.488	191.81	9.19	0.9
PRO	A	863	227.049	228.169	191.168	16.15	0.86
ASN	A	1074	184.757	221.391	154.156	17.15	0.85
LYS	A	417	214.635	190.144	255.125	19.88	0.77
GLN	A	787	232.766	223.886	167.537	16.9	0.74
ASP	A	985	220.054	222.41	236.626	9.42	0.71

TYR	A	396	187.54	191.018	242.224	14.07	0.68
ARG	A	1107	198.158	213.26	153.142	15.64	0.61
ARG	A	1039	208.399	212.127	174.062	18.66	0.6
LYS	A	1045	195.992	224.017	167.593	20.13	0.59
GLN	A	755	222.399	207.917	220.57	17.11	0.57
PHE	A	970	207.884	221.24	222.3	9.12	0.57
GLU	A	1031	214.171	212.613	172.972	16.07	0.57
SER	A	383	194.611	210.848	239	18.24	0.55
ARG	A	355	191.634	190.221	243.271	19.35	0.51
ASN	A	978	221.932	230.788	224.544	19.24	0.51
GLU	A	918	205.767	228.049	144.273	17.25	0.49
TYR	A	904	218.739	221.882	153.58	6.26	0.44
TYR	A	789	229.086	226.952	166.796	14.4	0.39
ASN	A	703	181.576	220.857	168.722	9.68	0.38
ILE	A	788	231.022	229.541	170.188	17.4	0.37
THR	A	547	183.854	208.339	225.799	17.16	0.36
ILE	A	896	222.096	228.806	156.893	15	0.36
GLU	A	1031	212.794	213.04	171.427	16.07	0.36
ALA	A	713	188.818	218.013	153.902	9.3	0.33
ARG	A	1039	208.63	212.25	171.784	9.42	0.33
ALA	A	890	227.156	214.702	165.465	17.04	0.31
PRO	A	1079	190.443	205.84	147.467	16.19	0.23
PHE	A	1121	200.522	203.821	144.984	14.32	0.23
TYR	A	707	184.817	210.012	157.821	14.63	0.22
THR	A	912	207.254	220.873	147.544	22.87	0.22
TYR	A	917	213.965	228.82	149.982	15.02	0.22
VAL	A	1040	200.536	215.385	168.426	24.07	0.22
GLN	A	1010	207.126	215.402	203.308	19.38	0.2
ASN	A	370	187.809	214.449	250.423	17.51	0.19
GLN	A	895	225.645	225.719	155.771	16.64	0.19
PHE	A	898	219.434	230.733	156.175	10.2	0.19
SER	A	1030	217.429	217.459	170.006	17.42	0.19
TYR	A	421	214.435	187.518	250.273	18.44	0.17
ASN	A	703	182.792	219.007	166.551	15.98	0.17
ALA	A	27	171.646	251.874	224.877	19.82	0
